# Relationship between serum uric acid levels and uric acid lowering therapy with the prognosis of patients with heart failure with preserved ejection fraction: a meta-analysis

**DOI:** 10.3389/fcvm.2024.1403242

**Published:** 2024-06-13

**Authors:** Linzhi Li, Ying Chang, Fei Li, Yuehui Yin

**Affiliations:** ^1^ Department of Cardiology, The Second Affiliated Hospital of Chongqing Medical University, Chongqing, China; ^2^ Department of Geriatrics, Chongqing General Hospital, Chongqing, China

**Keywords:** serum uric, uric acid lowering therapy, heart failure with preserved ejection fraction, meta-analysis, relationship

## Abstract

**Aims:**

This meta-analysis aimed to explore the association between serum uric acid levels and the efficacy of uric acid-lowering therapies on clinical outcomes among patients with heart failure with preserved ejection fraction (HFpEF).

**Methods:**

A comprehensive literature search was conducted through October 21, 2023, across PubMed, Embase, Cochrane Library, and Web of Science databases. The pooled effect sizes were estimated and presented with their respective 95% confidence intervals (CI). Subgroup analyses were conducted based on various factors, including sample size (<1,000 vs. ≥1,000), follow-up duration (<2 years vs. ≥2 years), study quality (assessed by a score of <7 vs. ≥7), ethnicity (Non-Asian vs. Asian), study design (prospective vs. retrospective), type of heart failure (HF) (acute vs. chronic), presence of hyperuricemia (yes or no), left ventricular ejection fraction (LVEF) thresholds (≥45% vs. ≥50%), and the type of uric acid-lowering therapy (traditional vs. novel).

**Results:**

The analysis included a total of 12 studies. Elevated serum uric acid levels were significantly linked to an increased risk of all-cause mortality [relative risk (RR): 1.21, 95% CI: 1.06–1.37, *P *= 0.004] and cardiovascular (CV) mortality (RR: 1.71, 95% CI: 1.42–2.04, *P *< 0.001) in HFpEF patients. Subgroup analyses confirmed this association, particularly in non-Asian populations, those with chronic HFpEF, and studies with a follow-up duration of two years or more. Additionally, higher uric acid levels were associated with an increased risk of HF-related hospitalization [hazard ratio (HR): 1.61, 95% CI: 1.12–2.34, *P* = 0.011]. Regarding treatment, uric acid-lowering therapy did not show a significant effect on reducing mortality in HFpEF patients. However, it was associated with a decreased risk of hospitalization due to HF (RR: 0.85, 95% CI: 0.79–0.91, *P* < 0.001).

**Conclusion:**

The findings of this study highlight the prognostic significance of serum uric acid levels in HFpEF and suggest that uric acid-lowering therapy may be beneficial in reducing the incidence of HF hospitalizations. Further research is warranted to elucidate the mechanisms by which uric acid-lowering therapy confers its potential benefits.

## Introduction

Heart failure (HF) is a clinical syndrome resulting from injury and congestion of the heart with a considerable rate of morbidity, mortality, poor functional capacity and quality of life, and high costs (1). Patients with HF with preserved ejection fraction (HFpEF) [left ventricular ejection fraction (LVEF) ≥50%] comprise nearly half of those with chronic HF (2). The incidence and prevalence of HFpEF continue to rise in tandem with the increasing age and burdens of obesity, sedentariness, and cardio metabolic disorders (3). HFpEF affects up to 32 million people worldwide (4). Functional capacity and quality of life are severely impaired in HFpEF, and morbidity and mortality are high (5). Patients with HFpEF are hospitalized approximately 1.4 times per year and have an annual mortality rate of approximately 15% (4). Effective treatments for HFpEF are still lacking (6, 7), despite the inhibitors of sodium-glucose transport protein 2 (SGLT2) inhibitors, which have demonstrated positive effects on the prognosis of HFpEF patients (8, 9). Therefore, it is of great significance for disease management to investigate the prognostic factors of patients with HFpEF.

Uric acid, the end-product of purine metabolism in humans, is not only a cause of gout, but also may play a role in developing cardiovascular diseases (CVD) (10, 11). A systematic review and meta-analysis published in 2021 indicated that serum uric acid is positively associated with the risk of adverse events in chronic HF patients (12). Another systematic review and meta-analysis demonstrated that every 1 mg/dl reduction in uric acid was associated with a significantly lower risk of a composite of cardiovascular (CV) death and hospitalization for HF (13). Previous systematic reviews or meta-analyses have focused on the relationship between uric acid levels and the prognosis of HF patients. There has not yet been a meta-analysis examining the association between uric acid levels and the outcomes for patients with HFpEF. In addition, several studies have found a relationship between uric acid-lowering therapy and prognosis in HF. A systematic review and meta-analysis of clinical studies found that uric acid-lowering treatments increased all-cause and CV mortality (14). In a recent meta-analysis, targeting uric acid-lowering did not improve the prognosis of patients with HF (15). In view of the conflicting results and the lack of meta-analysis on the outcome of uric acid-lowering therapy in patients with HFpEF, a meta-analysis is warranted.

Herein, this meta-analysis aims to investigate the relationship between serum uric acid levels and the therapeutic impact of uric acid-lowering therapy on the clinical outcomes of patients with HFpEF. This meta-analysis may contribute to the management of patients with HFpEF.

## Methods

This study followed the PRISMA (Preferred Reporting Items for Systematic Reviews and Meta-Analyses) guidelines (16).

### Search methods for identification of studies

From inception to October 21, 2023, PubMed, Embase, Cochrane Library, and Web of Science databases were searched. English search terms include “serum uric acid” AND “urate lowering drug” AND “heart failure”. The search strategy of the PubMed database was shown in Supplementary Material Table S1. The retrieved literature was imported into EndNote20, where an initial screening was conducted by reviewing the titles and abstracts. Following this preliminary assessment, full texts of the screened literature were read to exclude studies that did not meet the inclusion criteria. Subsequently, the remaining literature was incorporated into this study. Search strategies were methodically executed by two independent researchers (Linzhi Li and Ying Chang), with any arising discrepancies resolved through consultation with a third author (Fei Li).

### Eligibility criteria

Inclusion criteria were formulated based on the Population, Intervention, Comparator, Outcome, and Study design (PICOS) framework, encompassing: (1) P: patients with HFpEF; (2) I and C: serum uric acid levels/uric acid-lowering therapy; (3) O: all-cause mortality, CV mortality, HF hospitalization, and Kansas City Cardiomyopathy Questionnaire (KCCQ) clinical summary score; (3) S: cohort studies, and RCTs; (5) literature published in English.

Exclusion criteria: (1) animal experimental studies; (2) withdrawn studies; (3) reviews, meta-analyses, guidelines, consensus statements, errata, case reports, conference abstracts, editorial materials, letters, and trial registration records; (4) studies not relevant to the topic.

### Data extraction

Two reviewers (Linzhi Li and Ying Chang) independently collected data from the selected studies. Data extracted from the included studies encompassed the first name of the author, year of publication, country, study design, sample size, age (years), sample size of male, LVEF (%), definition of higher uric acid level (mg/dl), follow-up, and outcome. In instances of discrepancy, consensus was reached by referring to a third investigator (Fei Li) for arbitration.

### Assessment of quality of studies

The Newcastle-Ottawa Scale (NOS) (17) was used to assess the quality of cohort studies, with a total score of 9 points. Studies scoring 0–3 points were considered low quality, 4–6 points as medium quality, and 7–9 points as high quality. The Jadad scale (18) was utilized to evaluate the quality of RCTs, which comprises four items: generation of random sequences, allocation concealment, blinding (each item scoring up to 2 points), and withdrawals and dropouts (scoring 1 point). Studies scoring 1–3 points were deemed low quality, while those scoring 4–7 points were classified as high quality.

### Statistical analysis

All data were analyzed using Stata 15.0 software. For categorical variables, the relative risk (RR) or hazard ratio (HR) was used as the effect size, while for continuous outcomes, the weighted mean difference (WMD) was employed. The results of the combined effect size were presented with the effect size and its 95% confidence interval (CI). Heterogeneity tests were conducted for each outcome measure, with a random-effects model analysis being performed if *I*^2^ ≥ 50%, and a fixed-effects model analysis otherwise. Subgroup analyses were carried out based on sample size (<1,000, and ≥1,000), follow-up duration (<2 years, and ≥2 years), literature quality (<7, and ≥7), ethnicity (Non-Asian, and Asian), study type (prospective, and retrospective), type of HF (acute, and chronic), hyperuricemia (yes, or no), LVEF (≥45%, or ≥50%), and the type of uric acid-lowering therapy used (traditional, or novel). Sensitivity analyses were conducted for all outcomes. When ten or more studies were included for outcomes, publication bias was assessed with funnel plot. A *P* value of <0.05 was considered statistically significant.

## Results

### Study selection process and characteristics of included studies

**Figure 1:**
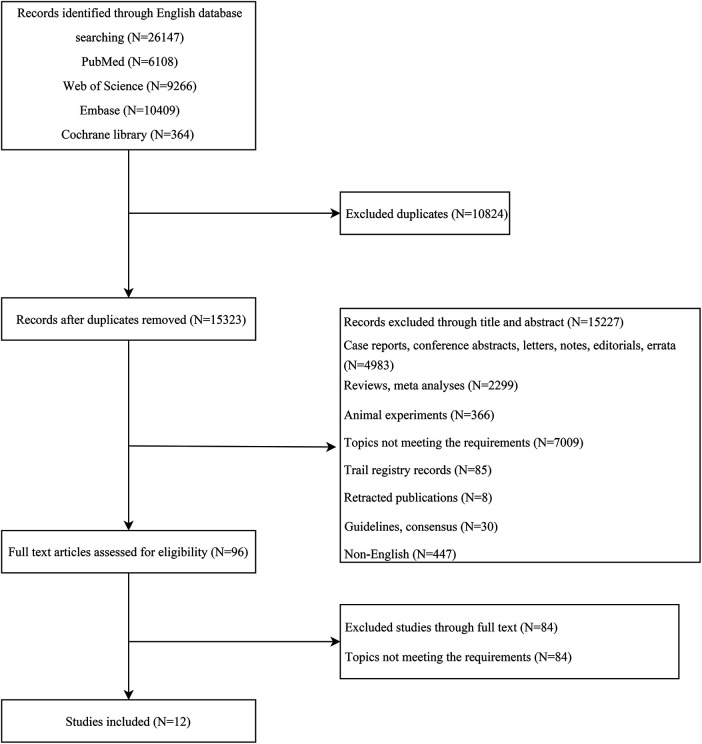
The flowchart of the literature screening process.

Initially, records were identified through various English databases: PubMed (*n* = 6,108), Web of Science (*n* = 9,266), Embase (*n* = 10,409), and the Cochrane Library (*n* = 364), totaling 26,147 records. A total of 10,824 duplicates were removed, leaving 15,323 records. These remaining records were then screened by title and abstract, resulting in the exclusion of 15,227 records. After exclusion, 96 full-text articles were assessed for eligibility. Finally, 12 (19–30) studies were included in the analysis. Figure 1 represents the literature screening process. The span of literature included in the study ranges from 2012 to 2023. Among the included articles, there were three RCTs and nine cohort studies. Six of the included articles were classified as high-quality studies. The shortest follow-up duration in the included literature was 12 weeks, while the longest was 4.81 years. Tables 1, 2 summarize the characteristics of the included studies.

**Table 1:** Basic information of literature on the association between uric acid and adverse outcomes of HF.

Author	Year	Country	Study design	Sample size	Age	Male，*n*	LVEF (%)	Definition of higher UA level (mg/dl)	Follow-up	Adjustment variables	Univariate, HR (95% CI)	Multivariable, HR (95% CI)	Outcome	NOS, Quality score
Shimizu	2015	Japan	Prospective cohort	424	68.36 ± 14.86	212	61.02 ± 9.10	7	897 days	Age, gender, systolic blood pressure, LVEF,B-type natriuretic peptide, presence of ischemic etiology, diabetes, atrial fibrillation, chronic kidney disease, anemia, hyperuricemia, and usage of blockers, renin-angiotensin-aldosterone system inhibitors, and diuretics	Cardiovascular mortality, 3.53 (1.35, 9.23); All-cause mortality, 2.08 (1.25, 3.46)	Cardiovascular mortality, 1.85 (0.58, 5.87); All-cause mortality, 1.98 (1.04, 3.79)	All-cause mortality Cardiovascular mortality	5
Selvaraj	2020	Multiple country	Prospective cohort	4,795	72.71 ± 8.45	2,316	58 ± 8	66–90 years, men 8 women 7.3 18–65 years, women 6.9	4 months	Age, sex, race, region, systolic blood pressure, heart rate, ejection fraction, NYHA class, history of HF hospitalization, duration of HF, atrial fibrillation, diabetes, body mass index, prior myocardial infarction, prior stroke, estimated glomerular filtration rate, haemoglobin, sodium, albumin, randomized treatment, diuretic use and NT-proBNP	Cardiovascular mortality, 1.71 (1.40, 2.09); All-cause mortality, 1.56 (1.33, 1.82); HF hospitalization, 2.05 (1.72, 2.45)	Cardiovascular mortality,1.58 (1.26, 1.98); All-cause mortality, 1.42 (1.18, 1.69); HF hospitalization, 1.61 (1.34, 1.94)	All-cause mortality Cardiovascular mortality HF hospitalization	7
Nishino	2022	Japan	Prospective cohort	464	81.81 ± 8.23	231	60.34 ± 7.85	7	480 days	NR	NR	NR	All-cause mortality HF hospitalization	6
Ambrosio	2021	Italy	Prospective cohort	4,938	64.6 (13.20)*	3,562	37.5 (13.48)	6.61	18 months	NR	NR	NR	Cardiovascular mortality HF hospitalization	7
Carnicelli	2020	USA	Retrospective cohort	7,004	68.16 ± 15.86	2,988	NR	6	2.6 years	Demographics (age, gender, and race),co-morbidities (diabetes, hypertension, and prior myocardial infarction), year of index echocardiogram (categorized), baseline measures (left ventricular ejection fraction, heart rate, systolic blood pressure, and body mass index), baseline medications (beta-blocker, angiotensin converting enzyme inhibitor, angiotensin receptor blocker, calcium channel blockers, and loop diuretic), and baseline laboratory measures (sodium, hemoglobin, blood urea nitrogen, and creatinine)	All-cause mortality, 1.24 (1.13, 1.36); HF hospitalization, 1.27 (1.17, 1.38)	All-cause mortality, 0.98 (0.89, 1.08); HF hospitalization, 1.03 (0.94, 1.12)	All-cause mortality HF hospitalization	6
Deng	2023	China	Prospective cohort	210	74 (67, 81)*	86	61 (58, 65)	7	278 days	Gender, NYHA class, coronary artery disease, atrial fibrillation, right ventricular dysfunction, NT-proBNP, and Cr	HF hospitalization, 2.98 (1.70, 5.21)	HF hospitalization, 3.03 (1.52–6.03)	HF hospitalization	7
Kobayashi	2020	Japan	Prospective cohort	516	78 ± 11	256	60 ± 8	7.4	749 days	Age, male, systolic blood pressure, sodium, log brain natriuretic peptide, albumin, blood urea nitrogen, use of diuretics before admission	NR	All-cause mortality, 1.23 (1.10, 1.39)	All-cause mortality	6
Wang	2023	China	Retrospective cohort	7,769	62.64 ± 15.12	6,244	NR	7	4.81 years	NR	NR	NR	All-cause mortality	5

HF, heart failure; USA, United States of America; NR, non-reported; LVEF, left ventricular ejection fraction; UA, uric acid; NYHA, New York Heart Association; NT-proBNP, N-terminal pro-B-type natriuretic peptide; Cr, creatinine; HR, hazard ratio; CI, confidence interval; NOS, Newcastle-Ottawa Scale.

*Stands for median (IQR).

**Table 2:** Basic information of literature on the association between uric acid-lowering therapy and adverse outcomes of HF.

Author	Year	Country	Study design	Sample size	Age	Male, *n*	Follow-up	Adjustment variables	Univariate, HR (95% CI)	Multivariable, HR (95% CI)	Outcome	NOS, quality score	Jadad, quality score
Málek	2012	Czech Republic	Prospective cohort	1,255	73.4 (45.7, 87.7)^a^	714	2 years	NR	NR	NR	All-cause mortality	6	
Solomon	2019	Multiple country	RCT	4,796	72.75 ± 8.40	2,317	35 months	NR	NR	NR	All-cause mortality Cardiovascular death HF hospitalization KCCQ-CS		4
Nassif	2021	Multiple country	RCT	324	70.35 ± 10.48	140	12 weeks	NR	NR	Cardiovascular mortality, 0.89 (0.70, 1.13); All-cause mortality, 1.02 (0.86, 1.21); HF hospitalization, 0.78 (0.64, 0.95)	KCCQ-CS		7
Nishino	2022	Japan	Prospective cohort	291	81.5 ± 7.33	146	480 days	NR	NR	NR	All-cause mortality HF hospitalization	6	
Anker	2022	Multiple country	RCT	4,005	72.8 ± 9.2	1,986	24 months	NR	NR	All-cause mortality, 1.63 (1.08, 2.45)	All-cause mortality Cardiovascular death HF rehospitalization		4

HF, heart failure; KCCQ-CS, Kansas City cardiomyopathy questionnaire clinical summary score; NT-proBNP, N-terminal pro B-type natriuretic peptide; NYHA, New York Heart Association; ^a^median (interquartile range); NR, non-reported; HR, hazard ratio; CI, confidence interval; NOS, Newcastle-Ottawa Scale.

### Meta-analysis of the association between serum uric acid level and all-cause mortality in patients with HFpEF

#### High serum uric acid level vs. low serum uric acid level (RR)

**Figure 2:**
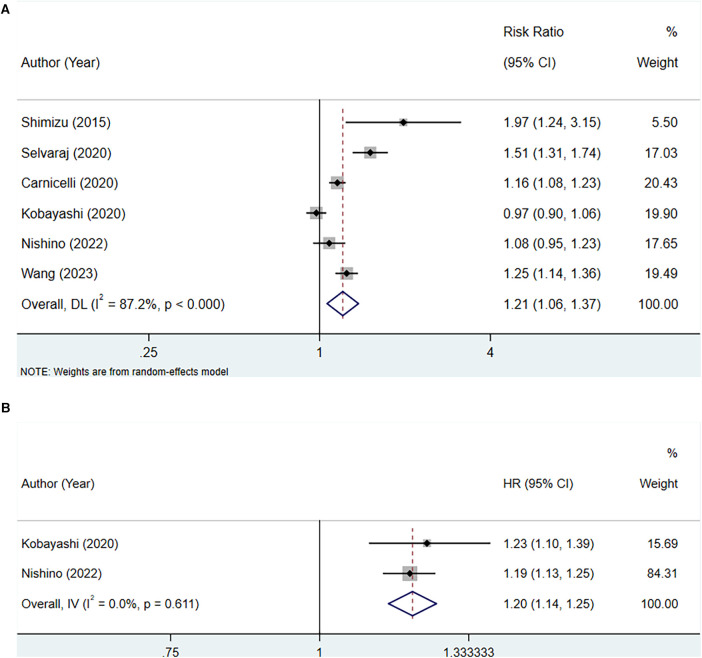
Meta-analysis of the association between serum uric acid level and all-cause mortality in patients with HFpEF; (**A**), high serum uric acid level; (**B**), per 1 mg/dl rise of serum uric acid level.

A total of six studies were included to assess the association between serum uric acid levels and all-cause mortality in patients with HFpEF. Due to high heterogeneity, as indicated by an *I*^2^ of 87.2%, a random-effects model was utilized for the analysis. The pooled analysis suggested that elevated serum uric acid levels were associated with an increased risk of all-cause mortality in patients with HFpEF (RR: 1.21, 95% CI: 1.06–1.37, *P *= 0.004) (Figure 2A, Table 3). The subgroup analyses showed that in studies with a sample size of 1,000 or greater (RR: 1.28, 95% CI: 1.12–1.46, *P* < 0.001), in studies with a follow-up duration of 2 years or longer (RR: 1.17, 95% CI: 1.01–1.35, *P* = 0.037), regardless of whether the quality score was less than 7 or 7 and above, among non-Asian populations (RR: 1.16, 95% CI: 1.08–1.24, *P* < 0.001), in retrospective study designs (RR: 1.19, 95% CI: 1.11–1.28, *P* < 0.001), and among patients with chronic HF (RR: 1.25, 95% CI: 1.14–1.37, *P* < 0.001), elevated serum uric acid levels were significantly associated with increased all-cause mortality in patients with HFpEF (Table 3).

**Table 3:** Meta-analysis of the association between serum uric acid level and outcomes in patients with HFpEF.

Outcomes	Indicators	RR/HR	*P*	*I* ^2^
Cardiovascular mortality (RR)	Overall	1.71 (1.42, 2.04)	<0.001	15.9%
All-cause mortality (RR)	Overall	1.21 (1.06, 1.37)	0.004	87.2%
	Sample size			
	<1,000	1.12 (0.913, 1.37)	0.279	79.3%
	≥1,000	1.28 (1.12, 1.46)	<0.001	82.3%
	Follow-up			
	<2 years	1.28 (0.92, 1.77)	0.144	87.0%
	≥2 years	1.17 (1.01, 1.35)	0.037	91.3%
	Quality score			
	<7	1.14 (1.02, 1.29)	0.025	82.9%
	≥7	1.51 (1.31, 1.74)	<0.001	0.0%
	Ethnicity			
	Non-Asian	1.16 (1.08, 1.24)	<0.001	0.0%
	Asian	1.16 (0.98, 1.38)	0.095	86.3%
	Study design			
	Prospective	1.26 (0.98, 1.60)	0.070	91.1%
	Retrospective	1.19 (1.11, 1.28)	<0.001	41.9%
	HF type			
	Acute	1.12 (0.91, 1.37)	0.279	79.3%
	Chronic	1.25 (1.14, 1.37)	<0.001	0.0%
All-cause mortality (HR)	Overall	1.20 (1.14, 1.25)	<0.001	0.0%
HF hospitalization (RR)	Overall	1.42 (0.97, 2.09)	0.070	97.9%
	Sample size			
	<1,000	1.03 (0.86, 1.23)	0.749	0.0%
	≥1,000	1.60 (0.99, 2.57)	0.053	98.5%
	Follow-up			
	<2 years	1.56 (0.95, 2.58)	0.082	95.4%
	≥2 years	1.12 (1.06, 1.18)	<0.001	0.0%
	Quality score			
	<7	1.11 (1.05, 1.17)	<0.001	0.0%
	≥7	1.99 (1.84, 2.16)	<0.001	0.0%
	Ethnicity			
	Non-Asian	1.39 (0.84, 2.32)	0.203	81.9
	Asian	1.03 (0.86, 1.23)	0.749	0.0%
	Study design			
	Prospective	1.56 (0.95, 2.58)	0.082	95.4%
	Retrospective	1.12 (1.06, 1.18)	<0.001	0.0%
HF hospitalization (HR)	Overall	1.61 (1.12, 2.34)	0.011	90.8%
	Sample size			
	<1,000	1.70 (0.58, 4.95)	0.332	89.4%
	≥1,000	1.60 (1.00, 2.56)	0.049	95.7%
	Follow-up			
	<2 years	1.80 (1.06, 3.07)	0.031	83.9%
	≥2 years	1.27 (1.17, 1.38)	<0.001	0.0%
	Quality score			
	<7	1.23 (1.06, 1.44)	0.008	18.9%
	≥7	2.24 (1.64, 3.06)	<0.001	36.1%
	Ethnicity			
	Non-Asian	1.27 (1.17, 1.38)	<0.001	0.0%
	Asian	1.70 (0.58, 4.95)	0.332	89.4%
	Study design			
	Prospective	1.80 (1.06, 3.07)	0.031	83.9%
	Retrospective	1.27 (1.17, 1.38)	<0.001	0.0%

HFpEF, heart failure with preserved ejection fraction; HF, heart failure; RR, relative risk; HR, hazard ratio.

#### Per 1 mg/dl rise of serum uric acid level (HR)

Two studies were included for analysis between every 1 mg/dl rise in serum uric acid level and all-cause mortality in patients with HFpEF. Heterogeneity testing yielded an *I*^2^ of 0.0%, thus a fixed-effect model was employed for the analysis. The pooled analysis demonstrated that for each 1 mg/dl elevation in serum uric acid levels, the risk of all-cause mortality increased (HR: 1.20, 95% CI: 1.14–1.25, *P* < 0.001) (Figure 2B, Table 3).

### Meta-analysis of the association between serum uric acid level and CV mortality in patients with HFpEF

#### High serum uric acid level vs. low serum uric acid level

**Figure 3:**
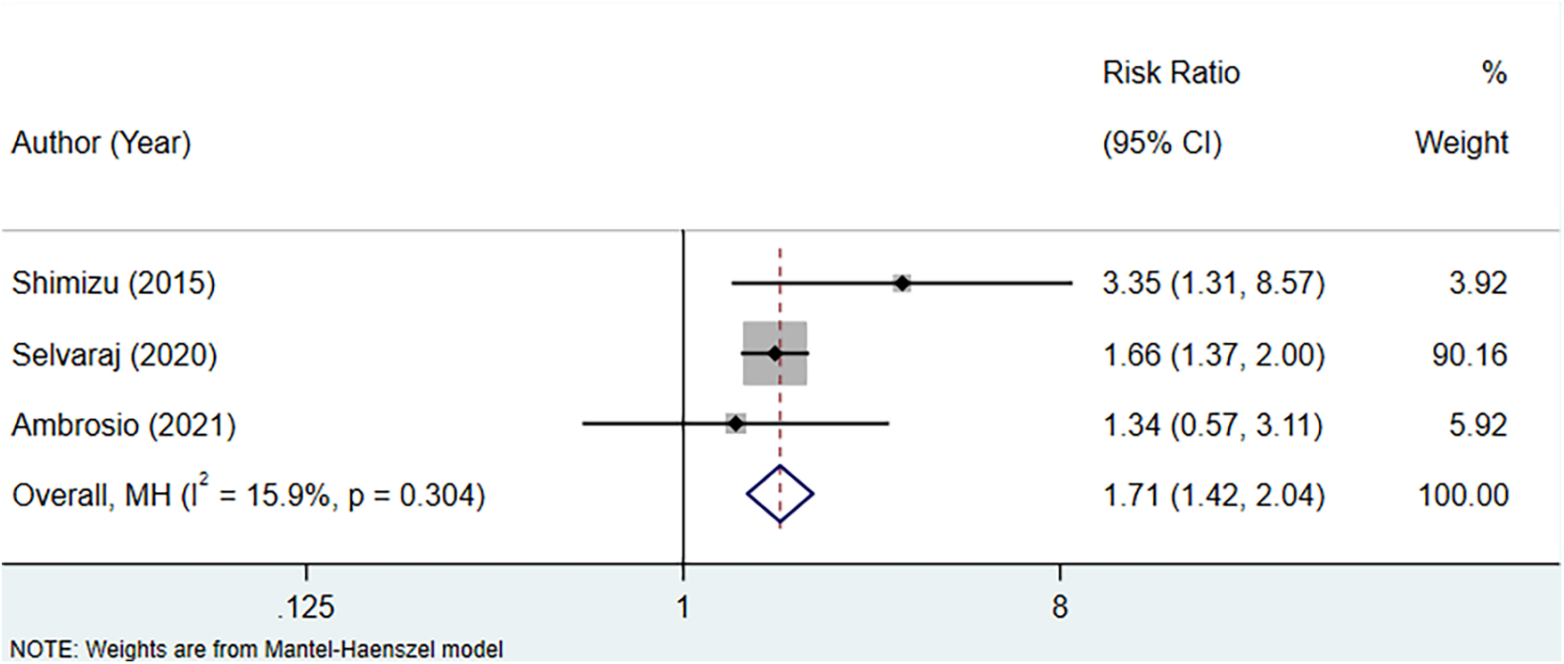
Meta-analysis of the association between serum uric acid level and CV mortality in patients with HFpEF.

Three studies were included to assess the association between serum uric acid level and CV mortality in patients with HFpEF. The fixed-effect model analysis suggested a significant association between increased serum uric acid levels and a higher risk of CV mortality (RR: 1.71, 95% CI: 1.42–2.04, *P* < 0.001) (Figure 3, Table 3).

### Meta-analysis of the association between serum uric acid level and HF hospitalization in patients with HFpEF

#### High serum uric acid level vs. low serum uric acid level (RR)

**Figure 4:**
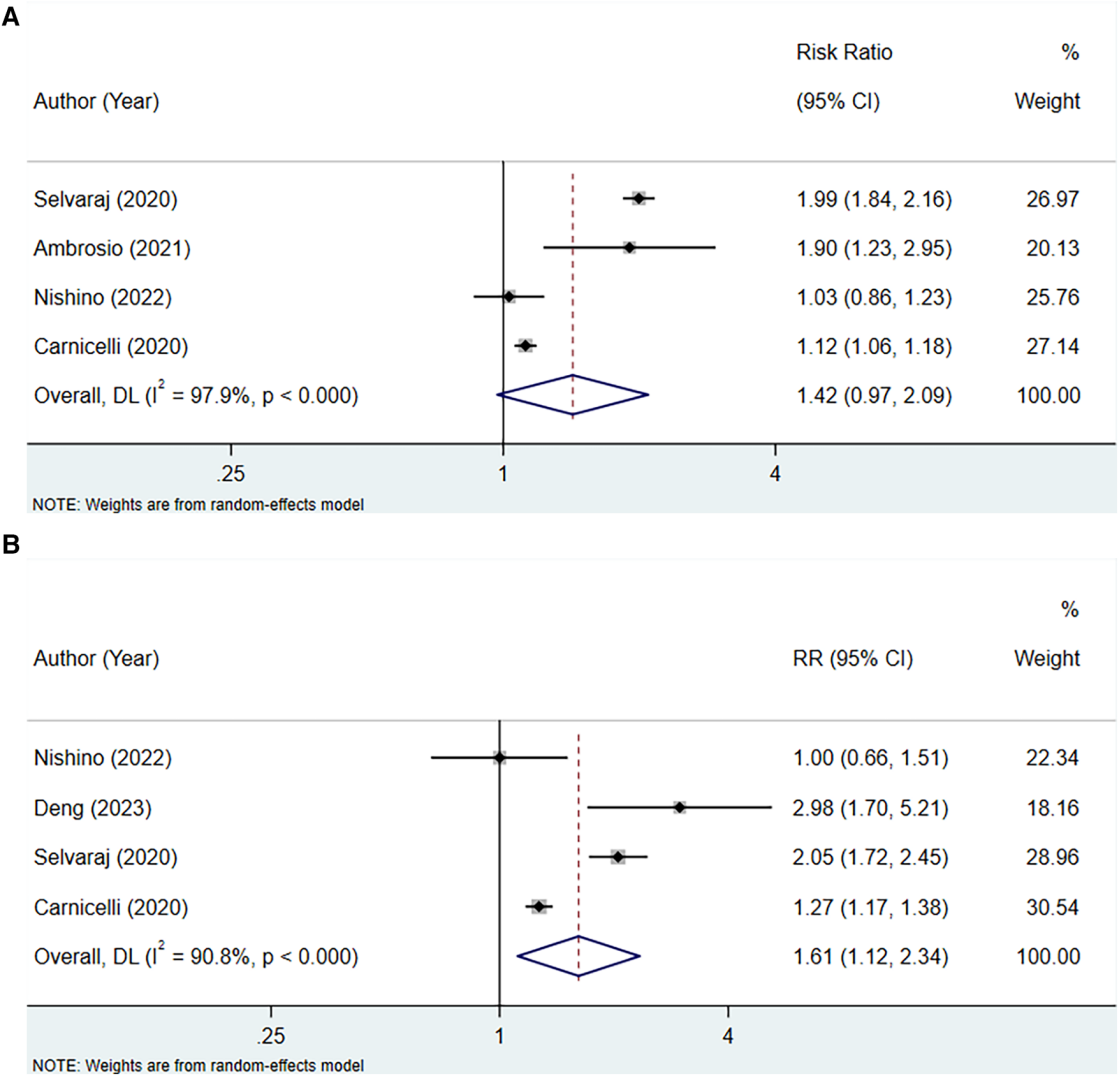
Meta-analysis of the association between serum uric acid level and HF hospitalization in patients with HFpEF; (**A**), RR; (**B**), HR.

Four studies assessed the association between serum uric acid level and HF hospitalization in patients with HFpEF, with heterogeneity testing showing an *I*^2^ of 97.9%. Consequently, analysis was conducted using a random-effects model. The outcome implied that elevated levels of serum uric acid did not have a significant correlation with HF hospitalization in patients with HFpEF (RR: 1.42, 95% CI: 0.97–2.09, *P* = 0.070) (Figure 4A, Table 3). However, subgroup analyses demonstrated that in contexts where the follow-up period extends to 2 years or longer, the studies were of a retrospective nature, and there was a notable association between increased uric acid levels and hospitalization for HF.

#### High serum uric acid level vs. low serum uric acid level (HR)

Four studies were included to examine the association between serum uric acid level (HR) and HF hospitalization in patients with HFpEF. The heterogeneity testing indicated an *I*^2^ of 90.8%, leading to the use of a random-effects model for analysis. The results suggested that an increase in uric acid levels was associated with an increased risk of hospitalization due to HF (HR: 1.61, 95% CI: 1.12–2.34, *P* = 0.011) (Figure 4B, Table 3). Subgroup analysis revealed that this relationship holds true across studies with a sample size of 1,000 or more, regardless of the duration of follow-up, the quality of the studies, whether the HF was acute or chronic, and also among non-Asian populations (Table 3).

### Meta-analysis of the association between uric acid-lowering therapy and all-cause mortality in patients with HFpEF

#### Experimental vs. control (RR)

**Figure 5:**
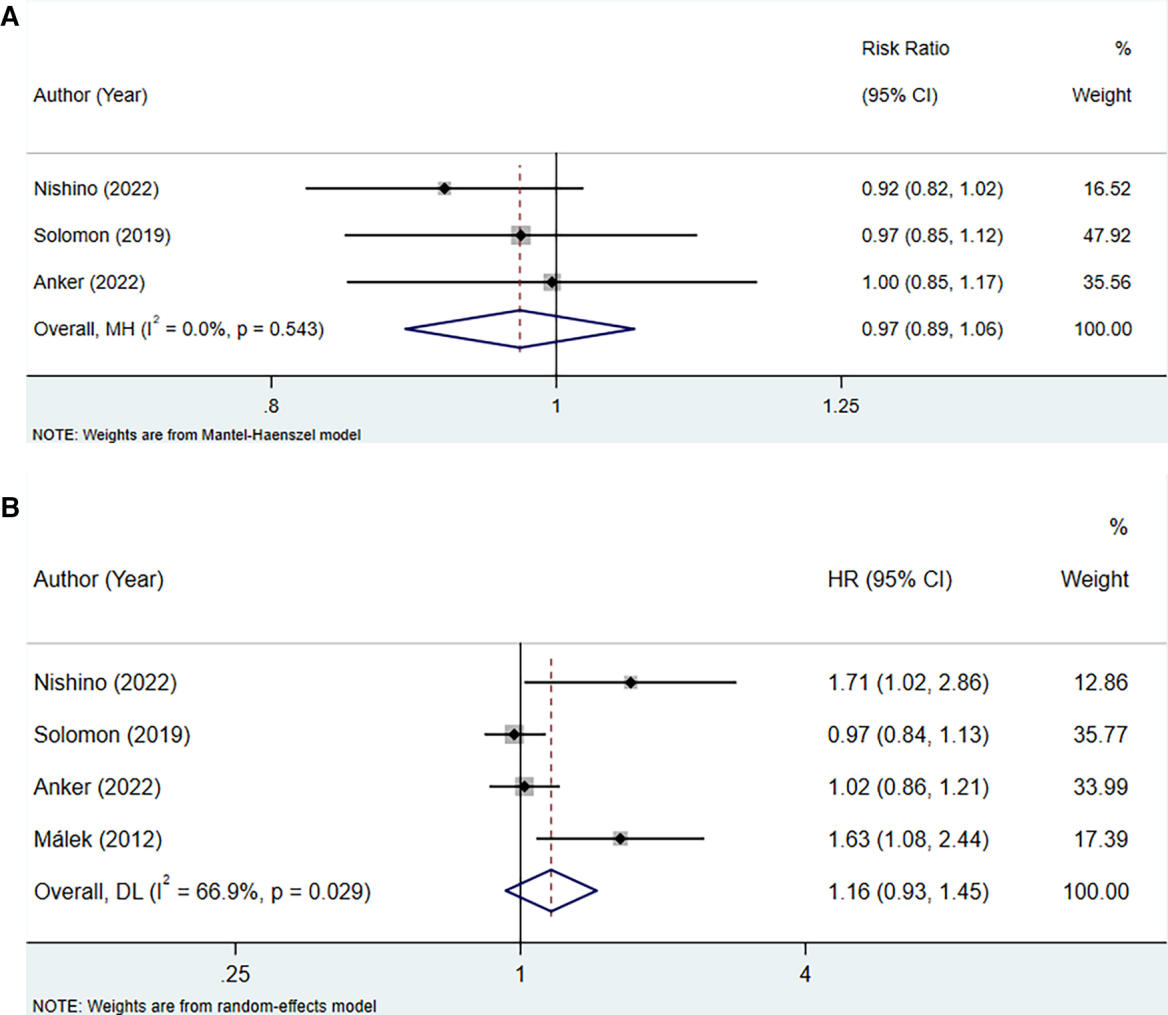
Meta-analysis of the association between uric acid-lowering therapy and all-cause mortality in patients with HFpEF; (**A**), RR; (**B**), HR.

The analysis incorporated three studies to assess the association between uric acid-lowering therapy and all-cause mortality in patients with HFpEF, revealing no heterogeneity (*I*^2^ = 0.0%), and hence, a fixed-effect model was utilized. The results showed that lowering uric acid levels through treatment did not significantly alter the outcome for all-cause mortality in patients with HFpEF (RR: 0.97, 95% CI: 0.89–1.06, *P* = 0.532) (Figure 5A, Table 4). This conclusion remained consistent across subgroups defined by the presence of hyperuricemia, LVEF, and the type of uric acid-lowering therapy used (Table 4).

**Table 4:** Meta-analysis of the association between uric acid-lowering therapy and outcome in patients with HFpEF.

Outcomes	Indicators	RR/HR/WMD	*P*	*I* ^2^
Cardiovascular mortality	Overall	0.92 (0.80, 1.07)	0.274	0.0%
All-cause mortality (RR)	Overall	0.97 (0.89, 1.06)	0.532	0.0%
	Hyperuricemia			
	No	0.98 (0.89, 1.09)	0.746	0.0%
	Yes	0.916 (0.82, 1.02)	0.115	0.0%
	LVEF			
	≥45%	0.97 (0.85, 1.12)	0.693	87.0%
	≥50%	0.97 (0.87, 1.09)	0.621	0.0%
	Uric acid-lowering drugs			
	Traditional	0.92 (0.82, 1.02)	0.115	0.0%
	Novel	0.98 (0.89, 1.09)	0.746	0.0%
All-cause mortality (HR)	Overall	1.16 (0.93, 1.45)	0.186	66.9%
	Hyperuricemia			
	No	1.24 (0.79,1.95)	0.351	76.6%
	Yes	1.17 (0.61, 2.26)	0.635	81.3%
	LVEF			
	≥45%	0.97 (0.84, 1.13)	0.687	0.0%
	≥50%	1.34 (0.92, 1.97)	0.128	71.1%
	Uric acid-lowering drugs			
	Traditional	1.66 (1.20, 2.28)	0.002	0.0%
	Novel	0.99 (0.89, 1.11)	0.877	0.0%
HF hospitalization	Overall	0.85 (0.79, 0.91)	<0.001	0.0%
	Hyperuricemia			
	No	0.85 (0.78, 0.92)	<0.001	0.0%
	Yes	0.83 (0.73, 0.94)	0.003	0.0%
	LVEF			
	≥45%	0.86 (0.79, 0.94)	<0.001	0.0%
	≥50%	0.81 (0.72, 1.18)	0.002	0.0%
	Uric acid-lowering drugs			
	Traditional	0.85 (0.78, 0.92)	<0.001	0.0%
	Novel	0.83 (0.73, 0.94)	0.003	0.0%
Change in KCCQ clinical summary score	Overall	1.96 (−0.91, 4.84)	0.181	60.3%

HFpEF, heart failure with preserved ejection fraction; HF, heart failure; RR, relative risk; HR, hazard ratio; WMD, weighted mean difference; LVEF, left ventricular ejection fraction; KCCQ: Kansas City cardiomyopathy questionnaire.

#### Experimental vs. control (HR)

The association between uric acid-lowering therapy and all-cause mortality in patients with HFpEF (HR) was analyzed in 4 studies. The heterogeneity testing resulted in an *I*^2^ of 66.9%, leading to the adoption of a random-effects model. The results indicated that uric acid-lowering therapy did not significantly reduce the risk of all-cause mortality (HR: 1.16, 95% CI: 0.93–1.45, *P *= 0.186) (Figure 5B, Table 4). Subgroup analysis revealed that only when uric acid-lowering medication was of the traditional type did the treatment increase the risk of all-cause mortality (HR:1.66, 95% CI: 1.20–2.28, *P *= 0.002) (Table 4).

### Meta-analysis of the association between uric acid-lowering therapy and CV mortality in patients with HFpEF

**Figure 6:**
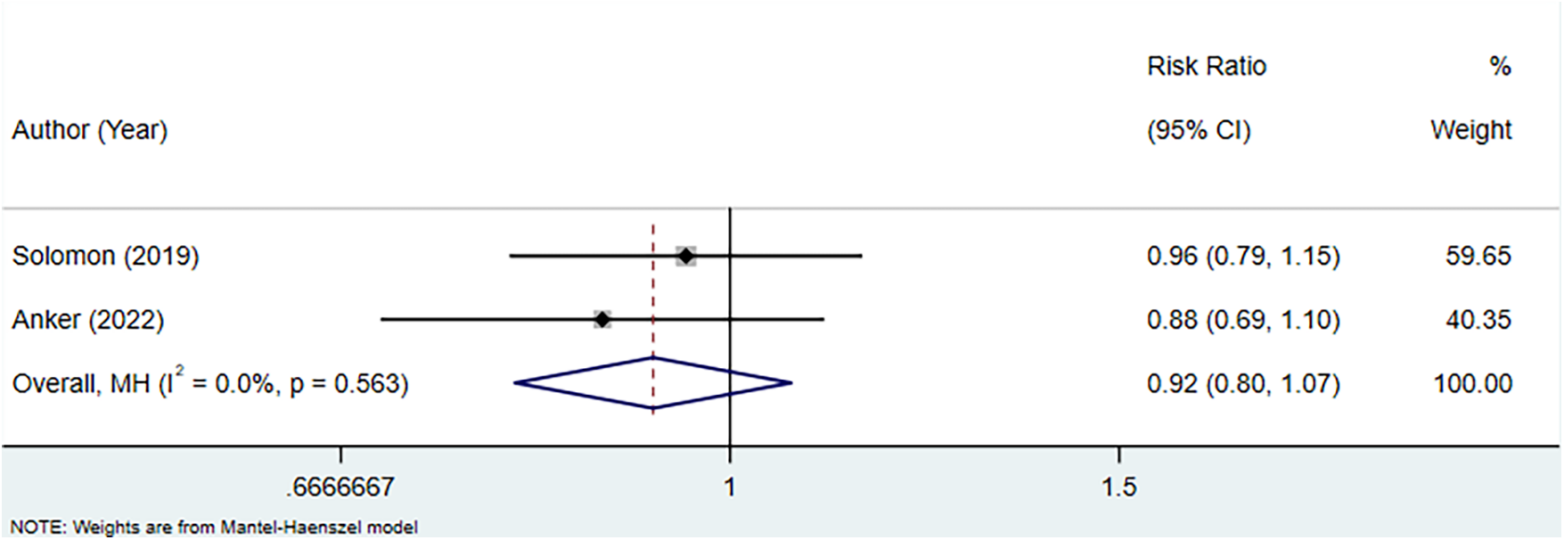
Meta-analysis of the association between uric acid-lowering therapy and CV mortality in patients with HFpEF.

Two studies were included to assess the association between uric acid-lowering therapy and CV mortality in patients with HFpEF. Heterogeneity testing showed an *I*^2^ of 0.0%, which led to the use of a fixed-effect model for analysis. The result indicated that uric acid-lowering therapy did not significantly improve the outcome of CV mortality (RR: 0.92, 95% CI: 0.80–1.07, *P *= 0.274) (Figure 6, Table 4).

### Meta-analysis of the association between uric acid-lowering therapy and HF hospitalization in patients with HFpEF

**Figure 7:**
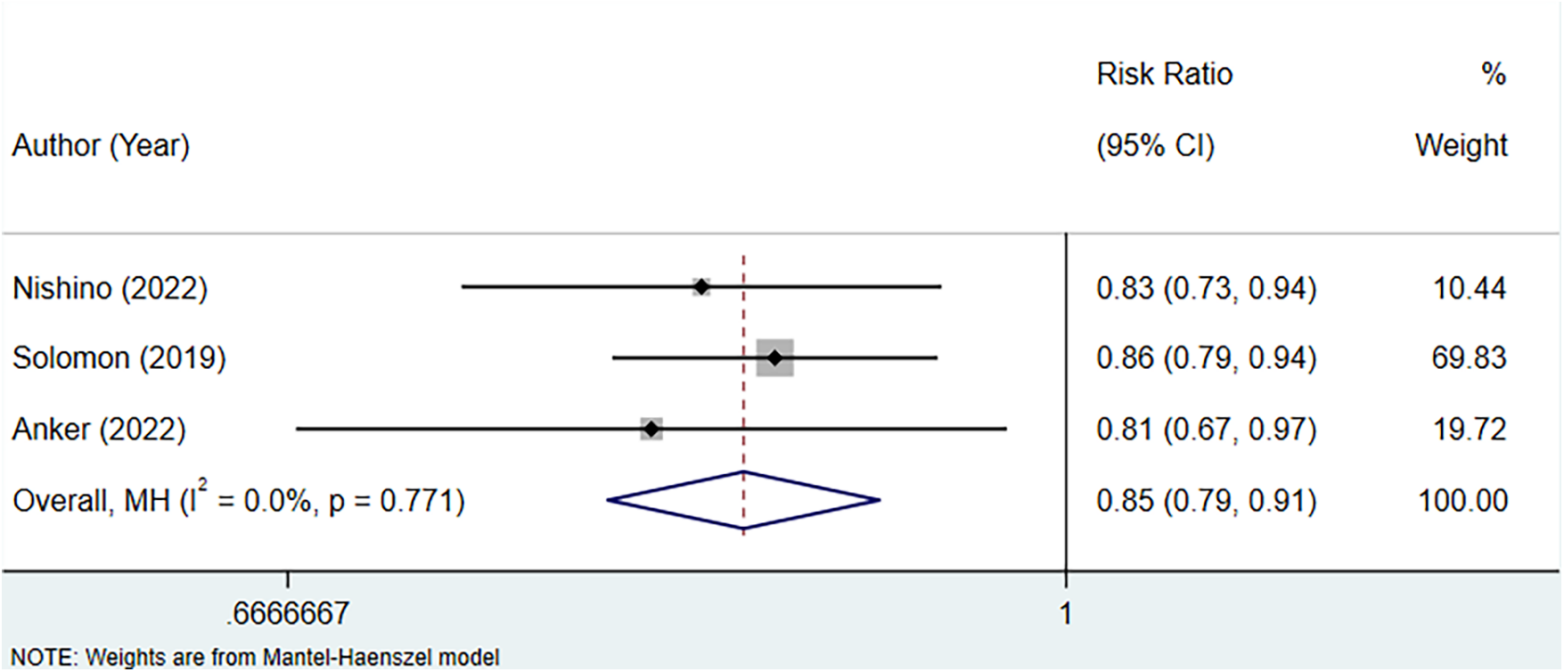
Meta-analysis of the association between uric acid-lowering therapy and HF hospitalization in patients with HFpEF.

A total of three studies were included to assess the association between uric acid-lowering therapy and HF hospitalization in patients with HFpEF. Heterogeneity testing revealed an *I*^2^ of 0.0%, hence a fixed-effect model was employed for analysis. The results suggested that uric acid-lowering therapy was associated with a lower risk of HF hospitalization (RR: 0.85, 95% CI: 0.79–0.91, *P *< 0.001) (Figure 7, Table 4). Subgroup analyses based on the presence of hyperuricemia, LVEF, and the type of uric acid-lowering therapy also yielded consistent results (Table 4).

### Meta-analysis of the association between uric acid-lowering therapy and change in KCCQ clinical summary score

**Figure 8:**
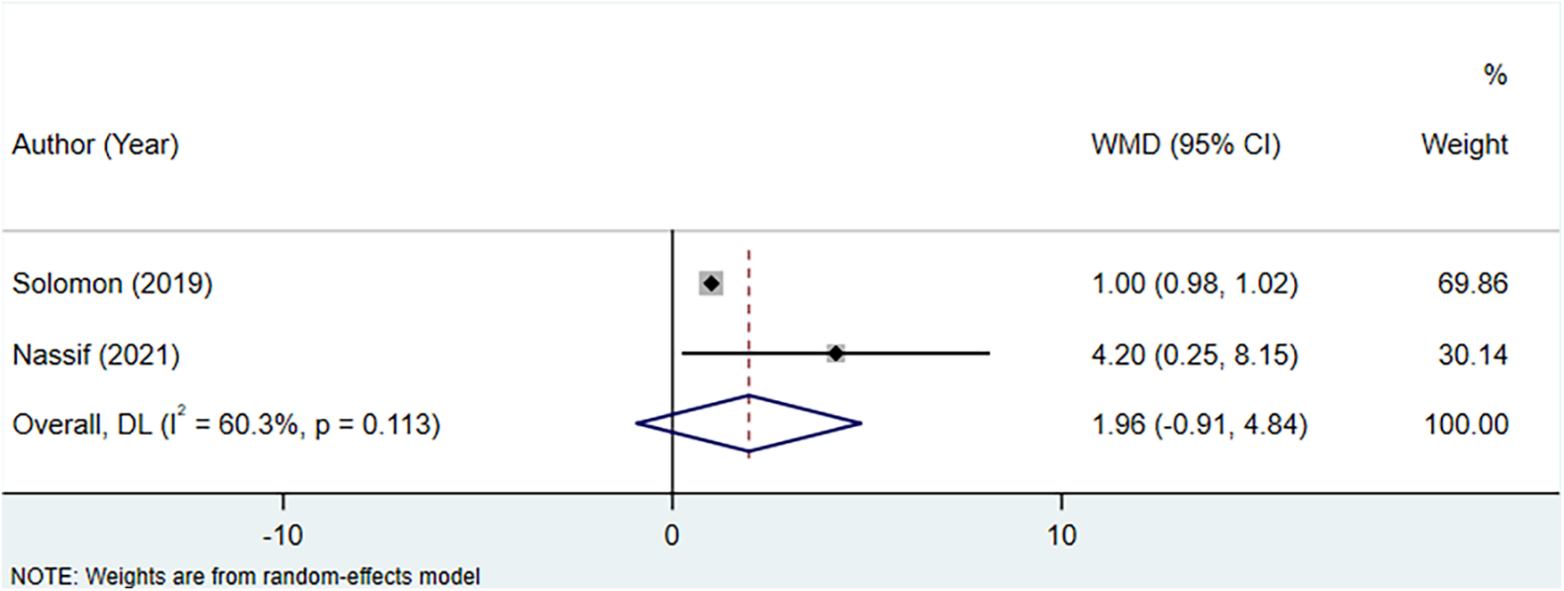
Meta-analysis of the association between uric acid-lowering therapy and change in KCCQ clinical summary score.

The analysis incorporated two studies to assess the association between uric acid-lowering therapy and change in KCCQ clinical summary score. Heterogeneity testing indicated an *I*^2^ of 60.3%, leading to the use of a random-effects model for the analysis. The results indicated that there was no significant difference in the change of the KCCQ clinical summary score between the uric acid-lowering therapy group and the control group (WMD: 1.964, 95% CI: −0.913 to 4.842, *P *= 0.181) (Figure 8, Table 4).

### Sensitivity analysis

Sensitivity analysis in our study demonstrated the robustness of our findings. By systematically excluding certain studies and re-evaluating the effect estimates, we have ensured that our conclusions were not unduly influenced by any single study or potential biases. This rigorous examination of our data strengthens the reliability of our results and provides confidence in the validity of our research (Table 4).

## Discussion

This study incorporated 12 eligible articles to separately explore the associations of serum uric acid levels and uric acid-lowering therapy with the prognosis of patients with HFpEF. The findings of this study reveal that elevated serum uric acid levels were consistently associated with an increased risk of all-cause mortality and CV mortality in HFpEF patients. Subgroup analyses further confirmed the association between serum uric acid levels and all-cause mortality, especially in non-Asian populations, those with chronic HFpEF, and when the follow-up duration was two years or longer. An increase in uric acid levels was also associated with an increased risk of hospitalization due to HF [hazard ratio (HR)]. In terms of therapeutic interventions, uric acid-lowering therapy did not significantly reduce mortality in patients with HFpEF. Nevertheless, uric acid-lowering therapy was associated with a reduced risk of HF hospitalization, indicating a potential benefit in managing this aspect of HFpEF prognosis.

Previous studies have illuminated that serum uric acid stands as a distinctive risk factor for HF and prognosis of HF (31–36). In a large Italian cohort, serum uric acid was an independent risk factor for all HF and fatal HF (31). A study conducted in Japan showed that elevated uric acid levels were associated with a higher incidence of the primary endpoint and rehospitalization owing to acute decompensated HF (32). Cicero et al. (33) found that hyperuricemia is an emerging risk factor in the pathogenesis of HF and is intricately linked to a bleaker prognosis in HF patients. In a meta-analysis, the author found that elevated serum uric acid levels independently predicted all-cause mortality and the combined endpoint of death or readmission in acute HF patients (34). In the study by Coiro et al., Elevated serum uric acid concentrations have incremental prognostic value in elderly patients with acute HF, regardless of etiology and systolic function (36). The result from a comprehensive meta-analysis revealed that higher serum uric acid levels were associated with an increased risk of all-cause mortality, cardiac death, and HF rehospitalization in HF patients (15). However, the prognostic value of serum uric acid level in patients with HFpEF has not been fully elucidated. Our findings indicate that elevated serum uric acid levels were associated with an increased risk of all-cause, CV mortality, and HF hospitalization in patients with HFpEF. In a study investigating whether serum uric acid level on admission could be associated with subsequent mortality in hospitalized patients with HFpEF, higher admission serum uric acid was an independent determinant of mortality in hospitalized HFpEF patients (25). In the study by Nishino et al. (21), uric acid was a predictor for the composite of all-cause death and HF re-hospitalization in patients with hyperuricemia and HFpEF. In hospitalized elderly patients with chronic HF, serum uric acid was an independent predictor of adverse outcomes, which can be seen in HFmrEF patients (37). In a study involving 210 patients with HFpEF, elevated serum uric acid was significantly associated with the HF readmission rate in patients with HFpEF (24). In a study conducted in China, uric acid level was associated with HF readmission in patients with HFpEF (38). Our findings suggest that elevated serum uric acid levels serve as a significant prognostic marker in patients with HFpEF, indicating a potential role in the pathophysiology of the disease and its clinical outcomes.

Several insights into the potential mechanisms by which lowering uric acid levels may improve outcomes in patients with HF were provided by previous studies. A systemic proinflammatory state induced by comorbidities, including hyperuricemia, could cause myocardial structural and functional alterations (39). Furthermore, elevated uric acid levels can trigger a systemic inflammatory response, predisposing to comorbidities such as infections and malignancies, which significantly contribute to the mortality rate in HFpEF (40). Elevated uric acid may lead to increased cytokine activation, insulin resistance and oxidative stress, impair endothelial function and activate the renin-angiotensin system (41–44). Uric acid may also directly contribute to HF worsening by elevating blood pressure (45), and reducing renal function (46).

In addition, HFpEF patients varied by LVEF have different clinical characteristics, prognosis, and treatment response (47, 48). In this study, HFpEF patients had differences in LVEF. Although the patients we included was mostly elderly, the age distribution was not uniform. Age-related mechanisms play an important role in the pathophysiology of HFpEF (49). Older patients are at higher risk of side effects from HF medications (50). The proportion of men and women in the studies we included was fairly evenly distributed. A previous study found that hyperuricemia was associated with HF readmission in all patients, especially men (24). This is consistent with the higher comorbidities burden in men with HF (51). The observed differences may suggest that the impact of serum uric acid levels in patients with HFpEF requires a more personalized approach, taking into account the specific conditions of each patient.

In this meta-analysis, the administration of uric acid-lowering therapy did not yield a statistically significant reduction in mortality rates among patients afflicted with HFpEF. However, it was observed that uric acid-lowering therapy was correlated with a decreased risk of HF hospitalization. A systematic review and meta-analysis of RCTs showed that uric acid-lowering therapies did not improve all-cause mortality and CV death in HF patients (52). A recent meta-analysis suggested that targeting uric acid-lowering as a therapeutic intervention did not improve the prognosis of patients with HF (15). However, a study enrolling patients with HFpEF from the Prospective Multicenter Observational Study of Patients With Heart Failure With Preserved Ejection Fraction (PURSUIT-HFpEF) registry suggested that comprehensive interventions for lowering uric acid, including the use of urate-lowering therapy, in patients with hyperuricemia and HFpEF can have an effect of beneficial prognosis (21). The conflicting results of these findings may be attributed to differences in uric acid-lowering therapies. In the study by Suzuki et al. (53), febuxostat was potentially more effective than allopurinol for treating patients with chronic HF and hyperuricemia. These findings suggest that while uric acid-lowering therapy may not improve mortality rates in HFpEF in this study, it could still play a role in managing the disease. Further research, including well-designed clinical trials, is needed to determine the impact of lowering uric acid therapies in patients with HFpEF.

The importance of our research lies in the following aspects. Firstly, this study underscores the importance of monitoring serum uric acid levels in patients with HFpEF as a marker for early identification of those at risk for adverse outcomes. By regularly assessing serum uric acid levels, clinicians can identify high-risk patients earlier and adjust treatment strategies promptly to prevent potential adverse outcomes. Secondly, the meta-analysis provides new guidance for clinical practice, suggesting that physicians should consider serum uric acid levels as a factor in management and treatment decisions for HFpEF patients. This may include more aggressive interventions for patients with hyperuricemia to mitigate the risk of adverse outcomes. Thirdly, the study also sets the stage for future research by exploring whether lowering serum uric acid levels could offer additional benefits for HFpEF patients and how personalized treatment plans can minimize the adverse outcomes of HF. This could involve larger-scale clinical trials to validate the efficacy of uric acid-lowering therapy and to determine the optimal therapeutic approaches and timing for such interventions.

The strengths of our study include several key aspects: Firstly, it is the first meta-analysis to explore the association between adverse outcomes and uric acid-lowering therapy in patients with HFpEF. Secondly, the study incorporated a substantial sample size from high-quality literature, which lends a degree of reliability and stability to the findings. Thirdly, subgroup analyses were conducted on outcomes related to all-cause mortality and HF hospitalization, stratifying the study population based on the presence of hyperuricemia, the defined range of LVEF, and the type of uric acid-lowering therapy used. However, there are limitations to consider: Firstly, initially, angiotensin receptor neprilysin inhibitors (ARNi) and SGLT2i are not specifically developed to lower uric acid levels. It is plausible that the amelioration of HF symptoms could secondarily result in decreased uric acid levels, as the resolution of HF can lead to improved renal function and reduced uric acid retention (54). Consequently, discerning whether the observed enhancements in HF prognosis are attributable to the direct pharmacological action of these drugs, or indirectly due to the amelioration of HF and its associated metabolic changes, remains a challenge. Secondly, there were different thresholds for defining hyperuricemia and hypouricemia across studies, which may influence the results and may be one of the sources of heterogeneity in the outcome measures. Thirdly, the limited number of studies reporting on certain outcomes may affect the stability of the findings. Fourth, our meta-analysis is unable to perform a formal assessment of publication bias using funnel plots for the outcomes evaluated. Commonly, funnel plots are utilized to detect potential publication bias when a sufficient number of studies—typically ten or more are available for an outcome. This graphical method aids in visualizing the distribution of study results and identifying any asymmetry that may suggest selective reporting or publication of results. In our analysis, each outcome was represented by fewer than ten studies, which limits the reliability of funnel plot analysis to detect publication bias. These limitations should be taken into account when interpreting the results and when designing future research to further investigate the role of uric acid levels and uric acid-lowering therapy in HFpEF patients.

## Conclusion

In summary, these results underscore the importance of serum uric acid levels in the prognosis of HFpEF and the potential utility of uric acid-lowering therapy in reducing HF hospitalization, while also highlighting the need for further research to clarify whether interventions targeting hyperuricemia can confer benefits to HFpEF patients.

## Data Availability

The raw data supporting the conclusions of this article will be made available by the authors, without undue reservation.
