# Supplementary material for: Relationship between serum uric acid levels and uric acid lowering therapy with the prognosis of patients with heart failure with preserved ejection fraction: a meta-analysis
Source: Front Cardiovasc Med. 2024 Jun 13;11:1403242. doi: 10.3389/fcvm.2024.1403242 (PMC11210376; doi:10.3389/fcvm.2024.1403242)
Supplement: Supplementary file 1 [file Table1.docx]

The PubMed database used the following terms: “Heart Failure” OR “Cardiac Failure” OR “Heart Decompensation” OR “Decompensation, Heart” OR “Heart Failure, Right-Sided” OR “Heart Failure, Right Sided” OR “Right-Sided Heart Failure” OR “Right Sided Heart Failure” OR “Myocardial Failure” OR “Congestive Heart Failure” OR “Heart Failure, Congestive” OR “Hert Failure, Left-Sided” OR “Heart Failure, Left Sided” OR “Left-Sided Heart Failure” OR “Left Sided Heart Failure” OR “Heart Failure, Diastolic” OR “Diastolic Heart Failures” OR “Heart Failure, Preserved Ejection Fraction” OR “Heart Failure, Normal Ejection Fraction” OR “Diastolic Heart Failure” OR “HFpEF” AND “uric acid” OR “UA” OR “Acid, Uric” OR “2,6,8-Trihydroxypurine” OR “Trioxopurine” OR “Potassium Urate” OR “Urate, Potassium” OR “Urate” OR “Ammonium Acid Urate” OR “Acid Urate, Ammonium” OR “Urate, Ammonium Acid” OR “Sodium Urate Monohydrate” OR “Monohydrate, Sodium Urate” OR “Urate Monohydrate, Sodium” OR “Monosodium Urate Monohydrate” OR “Monohydrate, Monosodium Urate” OR “Urate Monohydrate, Monosodium” OR “Sodium Acid Urate Monohydrate” OR “Sodium Urate” OR “Urate, Sodium” OR “Monosodium Urate” OR “Urate, Monosodium” OR “Sodium Acid Urate” OR “Acid Urate, Sodium” OR “Urate, Sodium Acid” OR “Hyperuricemia” OR “Gout” OR “Gouts” OR “Benzbromarone” OR “Benzbromaron” OR “Benzbromaron AL” OR “AL, Benzbromaron” OR “Benzbromaron-Ratiopharm” OR “Benzbromaron Ratiopharm” OR “Narcaricin” OR “Desuric” OR “Urinorm” OR “Acifugan” OR “Besuric” OR “Allopurinol” OR “Uribenz” OR “Allopurin” OR “Allorin” OR “Allpargin” OR “Allural” OR “Pan Quimica” OR “Apulonga” OR “Apurin” OR “Atisuril” OR “Bleminol” OR “Caplenal” OR “Capurate” OR “Cellidrin” OR “Embarin” OR “Suspendol” OR “Foligan” OR “Hamarin” OR “Lopurin” OR “Lysuron” OR “Jenapurinol” OR “Milurit” OR “Milurite” OR “Novopurol” OR “Uripurinol” OR “Urosin” OR “Urtias” OR “Xanthomax” OR “Uridocid” OR “Xanturic” OR “Zygout” OR “Zyloprim” OR “Zyloric” OR “Pureduct” OR “Purinol” OR “Progout” OR “Remid” OR “Rimapurinol” OR “Roucol” OR “Tipuric” OR “Allohexal” OR “Allohexan” OR “Alloprin” OR “Febuxostat” OR “TEI 6720” OR “6720, TEI” OR “TEI-6720” OR “TEI6720” OR “Uloric” OR “2-”3-cyano-4-isobutoxyphenyl-4-methyl-5-thiazolecarboxylic acid” OR “urate lowering drug” OR “ULT” OR “xanthine oxidase inhibitor” OR “Xanthine Oxidoreductase Inhibitor” OR “XO inhibitor” OR “XOI” OR “FYX-051” OR “topiroxostat” OR “3-”5-pyridin-4-yl-1H-”1,2,4 triazol-3-ylpyridine-2-carbonitrile” OR “Uricosuric Agents” OR “Agents, Uricosuric” OR “Uricosuric Agent” OR “Agent, Uricosuric” OR “Probenecid” OR “Probenecid Weimer” OR “Pro-Cid” OR “Probecid” OR “Benuryl” OR “Benecid” OR “Benemid” OR “Lesinurad” OR “““5-bromo-4-”4-cyclopropyl-1-naphthyl-4H-1,2,4-triazol-3-ylsulfanylacetic acid” OR “RDEA594” OR “Zurampic” OR “Verinurad” OR “2-”“3-”4-cyano-1-naphthyl-4-pyridinylsulfanyl-2-methylpropanoic acid” OR “RDEA3170” OR “Arhalofenate” OR “MBX-102” OR “Dotinurad” OR “FYU-981” OR ““3,5-dichloro-4-hydroxyphenyl-”1,1-dioxo-2H-1,3-benzothiazol-3-ylmethanone” OR “ARNi” OR “Angiotensin receptor neprilysin inhibitor” OR “sacubitril valsartan” OR “Angiotensin receptor neprilysin Inhibition” OR “LCZ 696” OR “LCZ696” OR “LCZ-696” OR “Entresto” OR “Sodium-Glucose Transporter 2 Inhibitors” OR “SGLT2i” OR “Sodium Glucose Transporter 2 Inhibitors” OR “SGLT-2 Inhibitors” OR “SGLT 2 Inhibitors” OR “SGLT2 Inhibitors” OR “Sodium-Glucose Transporter 2 Inhibitor” OR “Sodium Glucose Transporter 2 Inhibitor” OR “SGLT2 Inhibitor” OR “Inhibitor, SGLT2” OR “Gliflozins” OR “Gliflozin” OR “SGLT-2 Inhibitor” OR “Inhibitor, SGLT-2” OR “SGLT 2 Inhibitor” OR “Canagliflozin” OR “Invokana” OR “Canagliflozin Hemihydrate” OR “Canagliflozin, Anhydrous” OR “Dapagliflozin” OR “Farxiga” OR “Forxiga” OR “BMS 512148” OR “BMS512148” OR “BMS-512148” OR “Empagliflozin” OR “BI 10773” OR “BI10773” OR “BI-10773” OR “Jardiance” OR “ertugliflozin” OR “Ipragliflozin” OR “Licogliflozin” OR “LIK-066” OR “LIK066” OR “remogliflozin” OR “Sergliflozin” OR “Luseugliflozin” OR “sotagliflozin” OR “LX4211” OR “LX-4211”
